# Prescrição de antidepressivos na atenção primária: um estudo descritivo acerca da confiança dos profissionais médicos

**DOI:** 10.1590/0102-311XPT130323

**Published:** 2024-08-26

**Authors:** Hercules Fernandes Moreno, Amanda Cristina Galvão Oliveira de Almeida

**Affiliations:** 1 Universidade Federal da Bahia, Salvador, Brasil.

**Keywords:** Antidepressivos, Prescrições, Atenção Primária à Saúde, Antidepressive Agents, Prescriptions, Primary Health Care, Antidepresivos, Prescripciones, Atención Primaria de Salud

## Abstract

Os antidepressivos são a terceira classe medicamentosa mais prescrita, sendo que a maioria das prescrições não é realizada por especialistas. O objetivo do estudo é avaliar a autopercepção de confiança na prescrição de antidepressivos por médicos da atenção primária à saúde (APS). Foi realizado estudo de corte transversal de médicos atuantes na APS da cidade de Salvador, Bahia, Brasil. Foram excluídos psiquiatras ou residentes de psiquiatria. A autoavaliação da confiança, assim como a coleta de características dos participantes foi realizada por meio de questionário *online*. Variáveis categóricas foram descritas em termos de frequência absoluta e relativa. Variáveis contínuas foram descritas como média ou mediana, conforme distribuição de normalidade. No contexto total de 447 médicos, a amostra foi composta por 55 participantes. A média de idade foi de 37,2 (±12,8) anos. A maioria dos médicos (75%) reconheceu-se confiante na prescrição de antidepressivos. A autopercepção de confiança manteve-se predominante em cenários de pacientes idosos (69,2%) e portadores de comorbidades gerais (65,4%). A minoria mostrou confiança para prescrever antidepressivos a crianças/adolescentes (19,2%) e gestantes (26,9%). Para 80,4% dos participantes, os inibidores seletivos da recaptação de serotonina foram a classe farmacológica de maior confiança. O encaminhamento para o Centro de Atenção Psicossocial foi a estratégia mais referida em casos de insegurança na prescrição (32%). Até onde se sabe, esse é o primeiro estudo a abordar tal questão. Por essa razão, ele pode contribuir para a construção de ações de educação em saúde mais assertivas voltadas a médicos da APS.

## Abstract (en)

Antidepressants are the third most prescribed drug class, and most prescriptions are not performed by specialists. This study aimed to evaluate primary health care (PHC) physicians’ self-perceived confidence in prescribing antidepressants. This is a cross-sectional study with PHC physicians in the municipality of Salvador, Bahia State, Brazil. Psychiatrists or psychiatry residents were excluded. The self-assessment of confidence and the collection of participants’ characteristics was carried out by an online questionnaire. Categorical variables were presented in absolute and relative frequencies. Continuous variables were described as means or medians according to their normality distribution. Of 447 physicians, the sample consisted of 55 participants. Their mean age was 37.2 ± 12.8 years. Most physicians (75%) claimed confidence in prescribing antidepressants. Self-perceived confidence remained predominant in scenarios with older adults (69.2%) and patients with general comorbidities (65.4%). A minority showed confidence to prescribe antidepressants to children/adolescents (19.2%) and pregnant women (26.9%). For 80.4% of participants, selective serotonin reuptake inhibitors were the most trusted pharmacological class. Referral to the Psychosocial Care Center was the most reported strategy in cases of insecure prescription (32%). To the best of our knowledge, this is the first study to address such an issue. Thus, it can contribute to more assertive health education actions for PHC physicians.

## Resumen

Los antidepresivos son la tercera clase de medicamentos más prescritos, y la mayoría de las prescripciones no son realizadas por especialistas. El objetivo del estudio es evaluar la autopercepción de confianza en la prescripción de antidepresivos por parte de los médicos de atención primaria de salud (APS). Corte transversal de médicos que trabajan en la APS de la ciudad de Salvador, Bahia, Brasil. Se excluyeron a los psiquiatras o residentes de psiquiatría. La autoevaluación de la confianza, así como la recolección de las características de los participantes, fue realizada a través de un cuestionario online. Las variables categóricas se describieron en términos de frecuencia absoluta y relativa. Las variables continuas se describieron como media o mediana, según la distribución de normalidad. En el contexto total de 447 médicos, la muestra estaba formada por 55 participantes. La edad promedio fue de 37,2 (±12,8) años. La mayoría de los médicos (75%) se reconocieron confiados en la prescripción de antidepresivos. La autopercepción de confianza se mantuvo predominante en los escenarios de pacientes ancianos (69,2%) y portadores de comorbilidades generales (65,4%). La minoría mostró confianza para prescribir antidepresivos a niños/adolescentes (19,2%) y mujeres embarazadas (26,9%). Para el 80,4% de los participantes, los inhibidores selectivos de la recaptación de serotonina eran la clase farmacológica de mayor confianza. El encaminamiento para el Centro de Atención Psicosocial fue la estrategia más mencionada en los casos de inseguridad en la prescripción (32%). Hasta donde se sabe, este es el primer estudio que aborda este tema. Por esa razón, puede contribuir para la construcción de acciones de educación en salud más asertivas dirigidas a los médicos de la APS.

## Introdução

Os antidepressivos são a terceira classe medicamentosa mais prescrita e, entre os psicotrópicos, são os medicamentos mais utilizados, sendo que a maior parte dessas prescrições advém da atenção primária à saúde (APS) ^1^ ^,^ ^2^ ^,^ ^3^ ^,^ ^4^. Esse modelo de atenção, que visa a integralidade e a longitudinalidade do cuidado, é garantido inclusive para aquelas pessoas com demandas referentes à saúde mental ^5^ ^,^ ^6^, que muitas vezes podem representar mais de 30% das demandas na APS ^3^.

Em 2011, foi instituída no Brasil a Rede de Atenção Psicossocial (RAPS) que redefiniu os direitos e estabeleceu novas formas de manejo e alocação dos pacientes com transtornos mentais ^7^. Com isso, houve o estreitamento das relações entre a APS e os Centros de Atenção Psicossocial (CAPS) ^8^. No entanto, devido ao subfinanciamento crônico e às limitações estruturais do Sistema Único de Saúde (SUS), a oferta de pontos de atenção da RAPS, especializados e não especializados, ainda é insuficiente para atender a demanda populacional de cuidado em saúde mental ^9^ ^,^ ^10^. Nesse panorama, verifica-se que a superlotação, a desassistência e a sobrecarga de trabalho dos profissionais das unidades de atenção especializada, assim como pensamentos de impotência e de incapacidade dos profissionais das APS, resultam em prejuízo na qualidade do serviço prestado pela RAPS ^9^ ^,^ ^10^ ^,^ ^11^. Por essas razões, a APS precisa ser ratificada e instrumentalizada como a instância que pode garantir o principal cuidado à saúde mental da população, sobretudo quando se sabe da grande prevalência de transtornos ansiosos e que a depressão é a maior causa de incapacitação do mundo ^12^ ^,^ ^13^.

Na metade do século XX, foi descoberto o primeiro antidepressivo. No entanto, somente em meados dos anos 1980, a partir da introdução da fluoxetina no mercado, essa classe de fármacos se tornou mais popular, com satisfatório grau de segurança e tolerabilidade ^1^. Seu impacto foi tão expressivo que esse é o antidepressivo mais usado no Brasil até hoje ^2^ ^,^ ^4^. Entretanto, há significativa carência na oferta de antidepressivo com maior perfil de tolerabilidade no SUS, uma vez que a classe amplamente disponível é a de antidepressivos tricíclicos que, apesar de eficazes, têm efeitos adversos que, muitas vezes, limitam a sua prescrição ^14^.

A elevada frequência de prescrição de antidepressivos se deve a múltiplos fatores. Entre eles, cabe citar: (1) facilidade de uso; (2) satisfatória eficácia no controle de diversas condições, como os transtornos depressivos, os transtornos ansiosos, alguns transtornos alimentares, dor crônica e dependência de nicotina; (3) uso prolongado, geralmente por volta de um ano e meio; (4) baixa incidência de efeitos colaterais intensos e boa segurança, com exceção dos inibidores da monoamina oxidase (IMAO) e dos tricíclicos ^1^ ^,^ ^15^ ^,^ ^16^. Tendo em vista a sua ampla gama de indicações e elevado número de prescrições, é possível que os antidepressivos também sejam utilizados com outros objetivos além do tratamento de transtornos psiquiátricos no contexto da APS ^17^.

Diante disso, é relevante que todos os profissionais médicos, em especial aqueles que atendem nas unidades básicas de saúde (UBS) e nas unidades de saúde da família (USF), sintam-se confiantes e tenham capacitação técnica para prescrever antidepressivos, já que a maioria dessas indicações ocorrem na APS, ou seja, não são feitas por especialistas em saúde mental ^2^ ^,^ ^4^. Assim, uma formação adequada e projetos de educação médica continuada são essenciais para garantir que tais prescrições ocorram de forma precisa. Esse cenário torna-se ainda mais preocupante quando se vê a escassez de trabalhos publicados relativos à prescrição de antidepressivos na atenção primária, sobretudo quando esse fenômeno é estudado sob a ótica do médico prescritor ^18^.

Com base no exposto, o objetivo deste estudo é avaliar a autopercepção que os profissionais médicos da cidade de Salvador, Bahia, Brasil, têm acerca da confiança para prescrever antidepressivos na APS. Ademais, pretendeu-se verificar o nível de aptidão técnica e o papel da formação médica na confiança em prescrever tais medicamentos.

## Metodologia

### Desenho de estudo e amostra

Trata-se de um estudo observacional do tipo corte transversal que incluiu médicos atuantes na APS do SUS. O recrutamento foi realizado em ambiente virtual com disseminação do formulário pelo processo de *snowball* (bola de neve), haja vista a inviabilidade de contatar todos os médicos da rede. Os pesquisadores disponibilizaram o acesso ao questionário pelas redes sociais com solicitação de encaminhamento aos pares para os que finalizassem a resposta. O formulário foi elaborado pelos autores por meio da ferramenta Google Forms (https://workspace.google.com), amplamente utilizada por sua facilidade, aderência e segurança. O preenchimento foi individual, não supervisionado, em horário conforme a conveniência do participante. O período de coleta das respostas ocorreu entre março e setembro de 2022.

A população do estudo foi constituída por médicos não psiquiatras da APS em Salvador. Segundo o Cadastro Nacional de Estabelecimentos de Saúde do Brasil (CNES), em setembro de 2021, havia 447 médicos vinculados a esses estabelecimentos de saúde ^19^. Desse modo, assumindo um intervalo de 95% de confiança (IC95%), calculou-se um tamanho amostral necessário de 70 respondedores.

### Critérios de inclusão

Médicos atuantes em UBS ou USF na cidade de Salvador, independentemente de nacionalidade ou instituição de graduação, que concordaram em participar da pesquisa por meio do Termo de Consentimento Livre e Esclarecido (TCLE), entre março e junho de 2022.

### Critérios de exclusão

Foram excluídos do estudo médicos psiquiatras ou residentes em psiquiatria e aqueles com atuação em serviços especializados em saúde mental da RAPS.

### Dados coletados

A coleta foi realizada por questionário virtual. Foram coletados dados com o objetivo de caracterizar: (1) perfil sociodemográfico (idade, gênero e nacionalidade); (2) fluxo de encaminhamento dos pacientes com demandas em saúde mental; (3) formação médica (faculdade de graduação, tempo de atuação médica, percepção de qualidade da formação em psiquiatria e afinidade pela área); (4) autoavaliação de confiança na prescrição de antidepressivos (confiança em geral, em populações especiais, classes e fármacos de escolha); (5) avaliação de conhecimento básico acerca do manejo terapêutico. As questões referentes aos aspectos 3, 4 e 5 foram definidas como itens tipo Likert de 5 pontos (“concordo totalmente” a “discordo totalmente”).

### Plano de análise dos dados

Os dados coletados foram armazenados em banco de dados específico por meio do programa Excel, versão 2209 (https://products.office.com/). A análise estatística foi realizada no programa SPSS, versão 21.0 (https://www.ibm.com/).

Variáveis categóricas foram descritas em frequências simples e proporções (%). Variáveis quantitativas foram descritas como médias e desvios padrão (DP) ou mediana e intervalo interquartil (IIQ), conforme apropriado. A normalidade das variáveis quantitativas foi avaliada pelo teste estatístico de Shapiro-Wilk e pelas características da distribuição (*kurtosis* e *skewness*). A correlação entre as respostas do questionário foi realizada por meio do teste de Spearman, considerando significância estatística para um valor de p < 5%.

### Aspectos éticos

O estudo foi iniciado após a aprovação pela Secretaria Municipal de Saúde de Salvador e pelo Comitê de Ética em Pesquisa do Complexo Hospitalar Universitário Professor Edgard Santos, Universidade Federal da Bahia (CEP-HUPES/UFBA). Todos que aceitaram participar do estudo assinaram o TCLE elaborado para essa pesquisa.

## Resultados

### Características gerais da amostra

Setenta e sete médicos responderam ao formulário, dos quais um foi excluído por ter residência ou título de especialista em psiquiatria, quatro por atenderem em serviço especializado da RAPS e dezessete por não atenderem em UBS ou USF em Salvador. Um total de 55 participantes compuseram a amostra do estudo. A média de idade dos profissionais foi 37,2 (±12,8) anos. A maioria era do gênero feminino (52,7%), natural da Bahia (69,1%) e tinha quatro anos ou mais de formação médica (52,7%). Uma quantidade considerável dos participantes era residente/médico de Medicina da Família e Comunidade (40%) e/ou professor universitário/preceptor de residência de Medicina da Família e Comunidade (29%). As características gerais estão descritas na Tabela 1.

**Tabela 1:** Características gerais da amostra.

Variável	n/N	Total (%)
Idade (anos) [média ± DP]	55/55	37,2 ± 12,8
Gênero		
Masculino	26/55	47,3
Feminino	29/55	52,7
Naturalidade		
Bahia	38/54	70,4
Minas Gerais	7/54	13,0
Pernambuco	3/54	5,6
São Paulo	2/54	3,7
Outro estado	4/54	7,4
Faculdade da graduação		
Pública	20/54	37,0
Privada	28/54	51,9
Estrangeira	6/54	11,1
Tempo de educação médica (anos)		
≤ 1	10/55	18,2
1-2	5/55	9,1
2-3	7/55	12,1
3-4	8/55	14,5
≥ 5	25/55	45,5
Titulação acadêmica		
Residência médica	15/55	27,3
Especialização	8/55	14,5
Mestrado	3/55	5,5
Doutorado	3/55	5,5
Graduação	26/55	47,3
Área de residência médica		
Medicina da Família e Comunidade	15/55	27,3
Pediatria	4/55	7,3
Dermatologia	1/55	1,8
Clínica médica	1/55	1,8
Ginecologia e Obstetrícia	1/55	1,8
Medicina do Trabalho e Endocrinologia	1/55	1,8
Não fez/concluiu residência	32/55	58,2

DP: desvio padrão.

### Confiança na prescrição de antidepressivos

Cinquenta e dois participantes referiram prescrever antidepressivos na APS e foram incluídos na análise de confiança na prescrição. Para isso, foram classificados como confiantes os participantes que responderam “concordo totalmente” ou “concordo parcialmente”. O respectivo nível de confiança geral e em populações especiais está descrito na Tabela 2.

**Tabela 2:** Confiança na prescrição de antidepressivos.

**“*Eu me sinto confiante para prescrever antidepressivos*”**	Discordo totalmente	Discordo	Neutro	Concordo	Concordo totalmente
	n (%)	n (%)	n (%)	n (%)	n (%)
De modo geral	2 (3,8)	8 (15,4)	3 (5,8)	27 (51,9)	12 (23,1)
Populações especiais					
Crianças/Adolescentes	20 (38,5)	19 (36,5)	4 (7,7)	9 (17,3)	2 (3,8)
Idosos	6 (11,5)	12 (23,1)	1 (1,9)	27 (51,9)	9 (17,3)
Comorbidades gerais (ex.: hipertensão arterial sistêmica, diabetes mellitus)	5 (9,6)	8 (15,4)	8 (15,4)	28 (53,8)	6 (11,5)
Gestantes	25 (48,1)	15 (28,8)	1 (1,9)	10 (19,2)	4 (7,7)

Três a cada quatro (75%) médicos reconheceram-se confiantes na prescrição de antidepressivos. A autopercepção de confiança manteve-se predominante em cenários de pacientes idosos (69,2%) e portadores de comorbidades gerais (65,4%). A minoria dos profissionais mostrou confiança para prescrever antidepressivos às crianças (19,2%) e às gestantes (26,9%).

A classe de antidepressivos com maior perfil de confiança na prescrição médica foi dos inibidores seletivos da recaptação de serotonina (80,4%), seguido dos antidepressivos tricíclicos (11,8%). Os medicamentos que os médicos se sentiram mais confiantes em prescrever foram a fluoxetina (58,3%), escitalopram (20,8%) e sertralina (18,8%).

A correlação das respostas à afirmação “*Eu me sinto confiante para prescrever antidepressivos*” com as demais respostas do questionário foi apresentada na Tabela 3. A confiança do prescritor para pacientes em geral apresentou correlação forte (r > 0,5) com a confiança em crianças/adolescentes, idosos, pacientes com comorbidades gerais e gestantes (p < 0,001) e correlação moderada (r > 0,3) com a afinidade pela especialidade psiquiatria (p = 0,018). Não observamos correlação significativa da confiança na prescrição com conhecimento técnico ou confiança comparativa aos benzodiazepínicos.

**Tabela 3:** Correlação entre respostas do questionário e confiança do prescritor.

Declaração avaliativa	Coeficiente de Spearman	Coeficiente de determinação (rs²)	Valor de p
“*Antidepressivos são usados somente para depressão*”	0,127	0,02	0,360
“*Caso o primeiro antidepressivo prescrito na menor dose inicial não resulte em remissão dos sintomas alvo em 4-6 semanas, deve-se substituir o antidepressivo*”	-0,101	0,01	0,468
“*Os antidepressivos devem ser usados pelo menor período de tempo possível, geralmente inferior a seis meses, pois podem causar dependência química*”	-0,132	0,02	0,340
“*Eu me sinto confiante para prescrever antidepressivos para crianças/adolescentes*”	0,514	0,26	< 0,001
“*Eu me sinto confiante para prescrever antidepressivos para idosos*”	0,695	0,48	< 0,001
“*Eu me sinto confiante para prescrever antidepressivos para pacientes com comorbidades gerais*”	0,650	0,42	< 0,001
“*Eu me sinto confiante para prescrever antidepressivos para gestantes*”	0,541	0,29	< 0,001
“*Eu tenho afinidade pela especialidade psiquiatria*”	0,319	0,10	0,018
“*Eu me sinto mais confiante para prescrever benzodiazepínicos (ex.: clonazepam, diazepam) do que para antidepressivos*”	-0,089	0,01	0,524

No grupo sem confiança na prescrição de antidepressivos, observou-se maior frequência de médicos graduados em instituição privada (61,5% *vs*. 50%) e com tempo de atuação inferior a dois anos (30,8% *vs*. 15,4%). Nesse grupo, uma menor proporção de médicos tinha histórico de pós-graduação (30,8% *vs*. 49%). O grupo sem confiança tinha menor parcela de médicos de família e comunidade (23,1% *vs*. 30,8%) ou especialistas em outras áreas (7,7% *vs*. 12,8%).

A percepção de qualidade da formação acadêmica em psiquiatria foi semelhante nos dois grupos [mediana (IIQ): 3 (2-4) *vs*. 3 (2-3,5)]. Médicos sem confiança apresentaram tendência a menor afinidade com a especialidade psiquiatria [mediana (IIQ): 4 (3-5) *vs*. 3 (2,5-4,0)]. A comparação entre os grupos está descrita na Tabela 4.

**Tabela 4:** Comparação quanto à confiança na prescrição de antidepressivos.

Variável	Confiança na prescrição (N = 52)	
	Sim (n = 39)	Não (n = 13)
Idade (anos) [média ± DP]	35,9 ± 12,0	37,2 ± 12,8
Gênero masculino [n (%)]	20 (51,3)	5 (38,7)
Faculdade da graduação [n (%)]		
Pública	15 (38,5)	3 (23,1)
Privada	20 (51,3)	8 (61,5)
Estrangeira	4 (10,2)	2 (15,4)
Tempo de atuação médica (anos) [n (%)]		
≤ 1	6 (15,4)	4 (30,8)
2-4	14 (35,9)	5 (38,5)
≥ 5	19 (48,7)	4 (30,7)
Titulação acadêmica [n (%)]		
Graduação	16 (41,0)	9 (69,2)
Especialização	6 (15,4)	1 (7,7)
Residência médica	13 (33,3)	2 (15,4)
Mestrado	3 (7,7)	0 (0,0)
Doutorado	1 (2,6)	1 (7,7)
Área de residência médica [n (%)]		
Medicina da Família e Comunidade	12 (30,8)	3 (23,1)
Outra área	5 (12,8)	1 (7,7)
Não fez/concluiu residência	22 (56,4)	9 (69,2)
Qualidade da formação em Psiquiatria		
Nota [mediana (IIQ)]	3 (2-4)	3 (2,0-3,5)
Péssima (5)	6 (15,4)	1 (7,7)
Ruim (4)	5 (12,8)	4 (30,8)
Regular (3)	18 (46,2)	5 (38,5)
Boa (2)	10 (25,6)	3 (23,1)
Ótima (1)	0 (0,0)	0 (0,0)
“Eu tenho afinidade com a Psiquiatria” [n (%)]		
Escala likert, mediana (IIQ)	4 (3-5)	3 (2,5-4,0)
Discordo totalmente (1)	2 (5,1)	0 (0,0)
Discordo (2)	4 (10,3)	3 (23,1)
Neutro (3)	6 (15,4)	5 (38,5)
Concordo (4)	15 (38,5)	3 (23,1)
Concordo totalmente (5)	12 (30,8)	2 (15,4)

DP: desvio padrão; IIQ: intervalo interquartil.

Nota: na variável “tempo de atuação médica”, as subdivisões 1-2, 2-3 e 3-4 foram agregadas como “2-4”. Na variável “área de residência médica”, as subdivisões Pediatria, Dermatologia, Clínica Médica, Ginecologia e Obstetrícia, e Medicina do Trabalho e Endocrinologia foram agregadas como “Outra área”.

### Conhecimento técnico sobre antidepressivos

A maioria dos participantes discordou parcial ou plenamente com a declaração (abreviada) “*Antidepressivos são usados somente para depressão*” (85,5%). Acerca do padrão de manejo prático dos antidepressivos, os participantes também discordaram das seguintes afirmações: “*Caso o primeiro antidepressivo prescrito na menor dose terapêutica inicial não resulte em remissão dos sintomas alvo em 4-6 semanas, deve-se fazer a substituição por outro antidepressivo*” (77,8%); “*Os antidepressivos devem ser usados pelo menor período de tempo possível, geralmente inferior a seis meses, pois podem causar dependência química*” (74,1%). A Tabela 5 descreve as respostas obtidas.

**Tabela 5:** Conhecimento básico e manejo de antidepressivos.

Declaração avaliativa	Discordo totalmente	Discordo	Neutro	Concordo	Concordo totalmente
“*Antidepressivos são usados somente para depressão*”	39 (72,2)	8 (14,8)	1 (1,9)	6 (11,1)	0 (0,0)
“*Caso o primeiro antidepressivo prescrito na menor dose inicial não resulte em remissão dos sintomas alvo em 4-6 semanas, deve-se substituir o antidepressivo*”	29 (53,7)	13 (24,1)	2 (3,7)	6 (11,1)	4 (7,4)
“*Os antidepressivos devem ser usados pelo menor período de tempo possível, geralmente inferior a seis meses, pois podem causar dependência química*”	27 (50,0)	13 (24,1)	4 (7,4)	4 (7,4)	6 (11,1)

### Condução de caso em cenários de insegurança

Diante de situações em que não se sentem confiantes na prescrição de antidepressivos, diversas estratégias foram mencionadas. A maioria dos médicos afirmou conduzir o caso encaminhando para o CAPS (32%). A discussão do caso com colegas médicos também foi uma conduta prevalente nesse contexto, sendo preferencialmente com colega psiquiatra (27%) ou generalista mais experiente (18%), assim como com ex-professor de psiquiatria (9%). O apoio do Núcleo de Apoio à Saúde da Família (NASF) ganhou relevância na abordagem para 7% dos médicos participantes.

O perfil de pacientes encaminhados para atendimento especializado com psiquiatra foi representado como os casos de ideação suicida (31%), transtorno bipolar do humor (25%) e refratariedade ao tratamento com o medicamento prescrição habitual (24%). Entre outros motivos mencionados (3%), quase metade deles (45,4%) fez referência aos pacientes com resistência ao tratamento mesmo após a associação de antidepressivos.

## Discussão

### Proporção de prescritores

A APS tem um papel fundamental no que diz respeito ao tratamento de pessoas com transtornos mentais e, consequentemente, à indicação de uso de psicotrópicos, considerando-se que a maior parte das prescrições de antidepressivos advém desse nível de atenção à saúde ^2^ ^,^ ^4^. Nesse sentido, a alta proporção de médicos prescritores de antidepressivos observada nessa amostra (94,5%) está em concordância com o sugerido pela literatura atual ^20^ ^,^ ^21^. Meira et al. ^20^ observaram que, entre os usuários da APS, o consumo de todos os antidepressivos estudados mais que dobrou entre 2019 e 2020; já Matheus et al. ^21^ encontraram que 10% dos usuários da unidade retiraram algum antidepressivo das farmácias da APS entre 2014 e 2017. É possível sugerir que isso decorre do crescimento da incidência de condições clínicas tratáveis com antidepressivos e da facilidade de acesso a esses fárnacos ^22^ ^,^ ^23^.

Outra hipótese que pode explicar a elevada prevalência de médicos prescritores de antidepressivos é a problematização desenvolvida por Barbui & Garattini ^24^, ao relatarem que as prescrições dos médicos não psiquiatras são mais influenciadas pela publicidade da indústria farmacêutica do que pela evidência científica. Sendo assim, é possível também que ocorra a prescrição indiscriminada de antidepressivos ^24^.

### Confiança na prescrição

A maioria dos médicos que trabalham na APS declarou que se sente confiante em prescrever antidepressivos. Nessa circunstância, a despeito da literatura escassa acerca da confiança na prescrição de antidepressivos na APS, foi identificado um estudo qualitativo realizado na Irlanda que investigou a confiança dos médicos de família em descontinuar esses medicamentos. Nesse trabalho, a maioria dos médicos generalistas se sentia confiante em descontinuar o uso de antidepressivos ^25^. Além disso, um grupo de investigadores britânicos encontrou resultados que podem corroborar esses achados, ao investigar a opinião dos médicos da APS em relação ao uso de antidepressivos. Verificou-se que, apesar de algumas reservas, esses profissionais percebem a importância da prescrição desses medicamentos, desde que responsavelmente e baseado em critérios clínicos bem definidos ^26^.

Nessa perspectiva, a predominância da percepção de confiança observada neste estudo pode ser explicada pelo investimento do SUS em programas de residência médica e em capacitações profissionais. A exemplo disso, tem-se a implementação de mais vagas de residência em Medicina da Família e Comunidade e o incentivo financeiro governamental para esses residentes ^27^. O programa de residência em Medicina da Família e Comunidade tem como uma das suas propostas a capacitação dos médicos no manejo dos transtornos mentais comuns, referentes a problemas de saúde mental que não atingem os critérios diagnósticos para transtornos depressivos e ansiosos, mas que apresentam sintomas incapacitantes e que comprometem significativamente a qualidade de vida do paciente ^12^ ^,^ ^28^ ^,^ ^29^. Desse modo, conforme pontuado por Lecrubier & Hergueta ^30^, com a detecção efetiva dessas condições, não somente a adesão terapêutica aumenta, como também o tratamento se torna mais custo efetivo do ponto de vista da saúde pública.

### Confiança na prescrição para populações especiais

Neste trabalho, a maior parte da amostra relatou confiança em prescrever antidepressivos para idosos; contudo, existem especificidades importantes em se trabalhar com esse público que talvez estejam sendo desconsideradas e/ou negligenciadas. Conforme apresentado em outros trabalhos, o uso de antidepressivos em idosos é desafiador em razão de alterações nos processos farmacocinéticos, interações medicamentosas e comorbidades médicas gerais ^31^ ^,^ ^32^ ^,^ ^33^. Segundo observações preocupantes de Queiroz Netto et al. ^34^, as doses de antodepressivos prescritas para adultos e idosos eram semelhantes, sugerindo que os prescritores não se atentaram a questões particulares da farmacoterapia nessa faixa etária.

Uma consideração relevante é o aumento significativo do número de diagnósticos psiquiátricos e de prescrições de psicotrópicos para crianças e adolescentes ao longo dos anos. Essa tendência foi observada por diversos autores em diferentes países, como Reino Unido e Austrália ^35^ ^,^ ^36^ ^,^ ^37^. Como a demanda por serviços especializados é crescente e a disponibilidade é limitada, torna-se essencial que os médicos da APS sintam confiança para prescrever antidepressivos quando necessário. No entanto, como evidenciado pelos resultados deste estudo, a menor parte desses profissionais sente-se confiante em prescrever antidepressivos para crianças e adolescentes, o que pode representar um obstáculo significativo para o tratamento adequado de pessoas nessa faixa etária.

A minoria dos médicos prescritores estudados se sente confiante em indicar antidepressivos para gestantes. Tal fenômeno pode ser justificado quando se avalia a qualidade limitada das evidências científicas acerca dos efeitos teratogênicos e das demais complicações desses fármacos à mãe e ao feto ^38^ ^,^ ^39^. Entretanto, como apresentado por Nascimento et al. ^40^, 30% das gestantes brasileiras com diagnóstico prévio de depressão fizeram uso recente de medicamentos antidepressivos. Desse modo, apesar da incerteza acerca dos efeitos deletérios relacionados ao uso dos antidepressivos em mulheres grávidas, a alta prevalência de uso desses medicamentos e os riscos associados à falta de tratamento da depressão em gestantes tornam indispensáveis o domínio dos médicos da APS ^38^ ^,^ ^39^ ^,^ ^40^.

### Classes e medicamentos prescritos

Esse estudo encontrou resultados compatíveis com os já observados por outros autores no que se refere ao perfil de prescrição de antidepressivos no contexto brasileiro. Prevedello ^4^ e Braga et al. ^41^ verificaram que a classe antidepressiva mais prescrita era a de inibidores seletivos de recaptação de serotonina (ISRS). Outros artigos nacionais também chegaram à conclusão de que o fármaco mais prescrito é a fluoxetina ^2^ ^,^ ^42^. Dessa forma, como o único ISRS disponível nas farmácias das UBS e USF da cidade de Salvador é a fluoxetina, esse fármaco torna-se a primeira opção para a maioria dos profissionais ^43^.

### Estratégias de encaminhamento

O encaminhamento para os CAPS foi a estratégia mais referida pelos médicos em caso de falta de confiança na prescrição. Certamente, tais centros desempenham um papel crucial para os usuários do SUS com demandas em saúde mental. Isso se deve à possibilidade de um manejo mais adequado e amplo de pessoas com transtornos mentais graves e persistentes e de um acompanhamento interdisciplinar com profissionais especializados sem que o indivíduo perca o vínculo com a USF ou UBS de referência ^44^ ^,^ ^45^.

Nesse âmbito, a procura por aconselhamento de outros colegas médicos também foi citada em uma proporção significativa do estudo, achado compatível com Kelly et al. ^25^, já que alguns médicos de família também disseram procurar ajuda de colegas em caso de dúvidas. Outro ponto pertinente a ser abordado é a baixa proporção de médicos que solicitam apoio a equipe do NASF (7%). Esse programa foi criado em 2008 e, entre suas propostas, tem a intenção de garantir um suporte multiprofissional na APS, como a presença de psicólogos e/ou psiquiatras. Dada a sua importância no contexto da estratégia de saúde da família, parece que essa ferramenta está sendo subutilizada ou ainda pouco disponibilizada ^46^.

## Pontos fortes e limitações

Por fim, os pontos fortes deste trabalho concentram-se em seu ineditismo e originalidade. Até onde se tem conhecimento, esse é o primeiro estudo a investigar tal problemática no Brasil, tratando-se, então, de um estudo exploratório. No entanto, é importante pontuar as seguintes limitações: o desafio na construção do questionário, visto que não havia um instrumento de coleta validado que atendesse aos objetivos da pesquisa; viés de amostragem, ao ser composta por muitos professores e médicos de Medicina da Família e Comunidade, que podem ter mais facilidade em prescrever antidepressivos do que os médicos generalistas, sem especialização ou residência médica; tamanho amostral pequeno, restringindo a generalização dos resultados à população de médicos da APS; a incapacidade de fazer conclusões causais devido à sua natureza transversal; e, finalmente, o número limitado de trabalhos publicados acerca do tema, que impossibilitou um comparativo mais assertivo.

## Conclusão

O estudo revelou que a maioria dos médicos que atuam na APS sente-se confiante em prescrever antidepressivos, inclusive para idosos e pessoas com comorbidades médicas gerais. A exceção se faz para as populações pediátrica e de gestantes. Observou-se que, entre aqueles que não se sentem confiantes, a maioria se formou em instituições privadas, tem menos de dois anos de prática médica e não apresentam afinidade com a psiquiatria. Não foram encontradas associações com os conhecimentos técnicos. À luz desses achados, é necessária a realização de pesquisas mais amplas a fim de elucidar outros pontos-chave, entre eles a capacidade técnica desses profissionais em realizar a prescrição de antidepressivos. Assim, é preciso que intervenções sejam implementadas, como a adoção de programas de educação médica continuada voltados para a APS e a criação de cursos de capacitação em saúde mental, capazes de treinar esses profissionais para adotar condutas pautadas em evidências científicas e de prescrever medicamentos com base em diretrizes terapêuticas e protocolos clínicos.
